# Impact of Combined Light and Modified Atmosphere Packaging on Postharvest Quality and Carbohydrate Fluctuations of Kyoho Grapes

**DOI:** 10.3390/foods14193308

**Published:** 2025-09-24

**Authors:** Kunpeng Zhao, Shaoyu Tao, Zhaoyang Ding, Jing Xie

**Affiliations:** 1 College of Food Science and Technology, Shanghai Ocean University, Shanghai 201306, China; 2 Shanghai Engineering Research Center of Aquatic-Product Processing & Preservation, Shanghai 201306, China; 3 Marine Biomedical Science and Technology Innovation Platform of Lin-gang Special Area, Shanghai 201306, China

**Keywords:** Kyoho grapes, carbohydrate metabolism, modified atmosphere packaging, light treatment

## Abstract

Kyoho grapes are rich in nutrients, yet their susceptibility to spoilage poses a significant challenge for postharvest preservation. While light treatment can improve fruit quality and carbohydrate metabolism in postharvest grapes, the potential benefits of combining light treatment with modified atmosphere packaging (MAP) remain unexplored. A preservation method that combined red and blue light treatments with MAP has been developed to enhance postharvest fruit quality and carbohydrate metabolism in Kyoho grapes. Our study showed that this combined treatment significantly increased postharvest fruit hardness, as well as total soluble solids (TSS) and fruiting pedicel water content. It also improved the activities of superoxide dismutase (SOD) and phenylalanine ammonialyase (PAL) and increased the antioxidant, anti-browning capacity. This composite treatment slowed down sucrose decomposition by regulating the activities of key enzymes of carbohydrate metabolism (sucrose synthase (SS), sucrose phosphate synthase (SPS), neutral invertase (NI) and acid invertase (AI)). After 60 days of storage, the glucose, fructose, and sucrose contents of the RP group increased by 13.4%, 30.2%, and 18.1%, respectively, compared to the CK group (*p* < 0.05). In summary, light combined with modified atmosphere packaging significantly improved the physicochemical properties and sugar metabolism of postharvest grapes. The results indicated that the optimal treatment condition was continuous red-light irradiation combined with MAP. The hardness, TSS content, VC content and glucose content of Kyoho grapes in this treatment group were the best in all treatment groups.

## 1. Introduction

Kyoho grapes (Vitis Labrusca × Vinifera ‘Kyoho’), which originated in Japan, are bred by hybridizing different European and American grape varieties. These grapes are larger, with a purple-black color and a high sugar content ranging from 15% to 18% [1]. Grapes are abundant in nutrients, including sugars, organic acids, and phenolic compounds [2]. Kyoho grapes have a variety of health benefits and skin care properties, attracting consumers around the world [3]. As one of the most widely cultivated crops globally, grape production exceeded 79 million tons in 2018, according to the Food and Agriculture Organization (FAO) of the United Nations [4]. However, during postharvest storage, grapes are prone to rot and deterioration, including fruit softening and a reduction in sweetness [5,6]. The hardness and sucrose content of grapes are key factors that affect the quality of Kyoho grapes, influencing both their organoleptic quality and economic value. Numerous studies have demonstrated that carbohydrate metabolism and its related enzymes are closely linked to fruit quality during development [7]. Furthermore, the mechanisms of sugar accumulation and the roles of key enzymes can vary between different crops [8]. Therefore, investigating the regulation of glucose metabolism and the roles of key enzymes in Kyoho grapes could provide insights into the molecular basis of postharvest physiological changes, offering a theoretical foundation for optimizing fruit storage and preservation techniques [9].

Traditional postharvest grape preservation methods, such as chemical preservatives, UV-C, refrigeration, can prolong the shelf life of postharvest grapes to a certain extent [10,11,12]. However, these methods still have shortcomings, such as high cost and chemical residue. Research showed that grapes can remain fresh for 15 days when stored at 5 °C [13]. Compared to traditional preservation methods, red and blue light treatment offers several advantages, including the absence of chemical additives, low cost, non-toxicity, and no by-product residues [14,15]. Currently, red and blue light processing primarily utilizes light-emitting diodes (LEDs). LEDs have several benefits, such as high energy conversion efficiency, low surface temperatures, minimal heat production, compact size, and a long lifespan [16]. The spectral composition of LEDs can be precisely controlled, allowing them to adjust their wavelength according to the specific needs of plant photoreceptors. Because the spectral wavelength and luminous intensity of LED devices can be easily regulated, they are commonly integrated into other equipment such as refrigerators and preservation cabinets. The effectiveness of LED technology for extending postharvest shelf life has been well documented across various produce, particularly in strawberries [17] and cabbage [18].

Modified atmosphere packaging (MAP) can be classified into active and passive types. Active modified atmosphere packaging involves actively filling a gas mixture in specific proportions to alter the gas environment within the package, thereby preserving freshness [19,20]. Cefola and Pace proved that active modified atmosphere packaging with 20% O_2_ and 10% CO_2_ could effectively maintain the postharvest quality of grapes [21]. By comparison, passive modified atmosphere packaging relies on the respiration of fruits or vegetables to change the gas composition within the sealed package, thereby preserving freshness [22]. Hasan et al. highlighted the significant effect of MAP in preserving the freshness of persimmons [23]. Wang et al. combined melatonin with passive MAP to investigate the freshness of bamboo shoots. Their results showed that this combined treatment effectively extends the shelf life of postharvest bamboo shoots [24]. Among the environmental factors influencing grape berry quality and carbohydrate metabolism, light and CO_2_ play the most significant roles. Deng et al. showed that high concentrations of CO_2_ can effectively inhibit the activity of cellulase and reduce the shedding of fruit during storage [25]. Previous studies demonstrated that both regulated light and optimized packaging atmosphere composition can significantly enhance the postharvest longevity of fresh produce [26,27]. The combination of MAP and LED slightly increases the packaging cost of postharvest grapes. However, it can significantly improve the return on investment of postharvest grapes. Both technologies can be integrated into the existing packaging line infrastructure. There is still a lack of data on Kyoho grapes under LED + MAP conditions.

Our previous research has demonstrated that forced-air pre-cooling is the most effective method for pre-cooling grapes [28]. However, there is limited research on the postharvest effects of combining LED light with modified atmosphere packaging treatments for grapes. Therefore, this study aims to examine the impact of various treatments on the quality, nutritional value, and carbohydrate metabolism of grapes stored at low temperatures. This includes combining red and blue LED light treatments with modified atmosphere packaging, as well as applying light treatments alone.

## 2. Materials and Methods

### 2.1. Preparation of Samples and Experimental Design

105 kg (90 bunches of berries) of Kyoho grapes (*Vitis Labrusca* × *Vinifera* ‘*Kyoho*’), harvested from the Malu vineyard in Jiading, Shanghai (31°19′33” N, 121°16′27” E), were quickly transported to the laboratory. The grapes grew in fertile soil with plenty of sunshine and abundant precipitation. The peel color of Kyoho grape was deep purple, which ensured the high consistency of physiological and edible maturity. The Kyoho grapes with deep purple in color and uniform in size and quality were selected for the study. After pre-cooling with strong air flow, the berries were randomly divided into five groups. Each treatment group contained a total of 18 bunches of berries. Three bunches of grapes were prepared at each storage time point. Then the grapes were stored in a refrigerator at −1 °C under fixed LED lights (LS-T5Y-5W-A, Blue Shark Lighting Co., Ltd., Zhongshan, China), based on the different light treatment protocols outlined in Table 1. The active modified atmosphere packaging used had a composition of 3% O_2_, 15% CO_2_, and 82% N_2_. The modified atmosphere packaging bags used were a nylon packaging bag provided by Shenzhen Xizhilong Packaging Products Co., Ltd. (Shenzhen, China), with a size of 25 × 30 cm and a thickness of 0.32 mm. Each MAP bag contained a bunch of grapes. The bag-type modified atmosphere lock fresh packaging machine (MAP-JY500D, Shanghai Jiyi Machinery Co., Ltd., Shanghai, China) was used for modified atmosphere packaging. Sunlight-proofing was achieved by placing Kyoho grapes packaged in modified atmosphere packaging bags into a refrigerator without LED light to achieve complete avoidance of light. The CK, BK and RK groups were packed in the same material as the treatment group, but not sealed. The relative humidity was 75% during storage, and there was no background light. Relevant quality parameters were measured every 10 days.

### 2.2. Visual Quality Assessment

The visual appearance of grape samples was documented photographically at each sampling interval. All photos were taken by mobile phone (iPhone 14 pro, foxconn technology group, Zhengzhou, China).

### 2.3. Determination of Hardness, Electrical Conductivity and Malondialdehyde (MDA)

Grape berries were placed under a 2 cm diameter cylindrical probe for texture testing using a texture analyzer (TA-XT Plus C, Stable Micro Systems Co., Ltd., Surrey, UK). The test parameters were: fruit crush mode with a starting force of 0.038 N, a deformation of 30%, a test speed of 30 mm/s and a return distance of 15 mm.

Electrical conductivity was determined by referring to the method proposed by Liang et al. [29] with slight modifications. 2 g of round fleshy grape slices (5 mm in diameter) were extracted. The samples were placed in test tubes containing 30 mL of distilled water. Initial conductivity (E1) was determined by digital conductivity meter (DDS-307A, INESA, Shanghai, China). After standing for 2 h, the final conductivity (E2) was determined by boiling again for 20 min and cooling to room temperature. Relative electrolyte leakage was calculated by the following equation:

(1) $$\mathrm{Relative}\;\mathrm{electrolyte}\;\mathrm{leakage}\;(\%)=(\frac{E1}{E2})\times 100\%$$

The MDA content was determined by reaction with thiobarbituric acid and absorbance at 532 nm using the MDA assay kit (Solarbio Science & Technology Co., Ltd., Beijing, China). Perform absorbance measurements using an enzyme calibration (Spark, TECAN, Shanghai, China).

### 2.4. Determination of Vitamin C (VC), Fruiting Pedicel Water Content and Total Soluble Solid (TSS)

VC was measured using the assay kit (Nanjing Jiancheng Bioengineering Institute, Nanjing, China).Grape berries were cut into equal lengths using a sterile blade, and the water content of the pedicels was measured using a moisture tester (HX-Q10, HUXI, Shanghai, China). The experimental method of Pérez-Pérez et al. was referenced and improved [30]. A 5 g sample of grapes was taken, ground thoroughly, and one drop of the supernatant was used to determine the TSS mass fraction using a refractometer (PAL-1, ATAGO, Tokyo, Japan).

### 2.5. Determination of Polyphenol Oxidase (PPO), Superoxide Dismutase (SOD) and Phenylalanine Ammonia-Lyase (PAL)

PPO, SOD, and PAL activities were determined using the Activity Assay Kit (Solarbio Science & Technology Co., Ltd., Beijing, China). SOD activity was measured using the WST-1 method. The reagents were added and mixed thoroughly according to the instructions, followed by incubation in a 37 °C water bath (J-HH-6A, Guansen Biotechnology Co., Ltd., Shanghai, China) for 30 min. The absorbance of the reaction solution was then measured at 450 nm. Total SOD activity was expressed as U/g. PAL catalyzes the conversion of L-phenylalanine to trans-cinnamic acid and ammonia. Since trans-cinnamic acid has a maximum absorbance at 290 nm, PAL activity was calculated by measuring the rate of increase in absorbance. PPO catalyzes the oxidation of catechol to form catechol quinone, which has a characteristic absorbance at 410 nm.

### 2.6. Determination of Neutral Invertase (NI) and Acid Invertase (AI)

The activities of NI and AI enzymes were determined by using the NI and AI Enzyme Activity Assay Kit (Solarbio Science & Technology Co., Ltd., Beijing, China). Firstly, NI and AI enzymes catalyzed the decomposition of sucrose to produce reducing sugar, which subsequently reacted with 3,5-dinitrosalicylic acid to produce a brown-red amino compound. The compound has a characteristic light absorption peak at 540 nm. The rate of increase in light absorption at 540 nm is proportional to the enzyme activity within a certain range. By determining the rate of change in light absorption, the activity of NI and AI enzymes can be determined.

### 2.7. Determination of Sucrose Synthase (SS) and Sucrose Phosphate Synthase (SPS)

SS and SPS enzyme activities were determined using the SS and SPS Enzyme Activity Assay Kit (Solarbio Science & Technology Co., Ltd., Beijing, China). First, SS and SPS enzymes catalyze the reaction of the corresponding substrates to produce sucrose or sucrose phosphate, which subsequently reacts with resorcinol to produce a color change that has a characteristic absorbance peak at 480 nm. Enzyme activity is directly proportional to color shade, and SS and SPS enzyme activity can be determined by measuring the absorbance change or rate of change.

### 2.8. Determination of Glucose Content, Fructose Content and Sucrose Content

Glucose, fructose and sucrose contents were measured using the Beijing Solarbio Science & Technology Co., Ltd. Assay Kit (Solarbio Science & Technology Co., Ltd., Beijing, China). Glucose oxidase was used to catalyze the oxidation of glucose to produce gluconic acid and hydrogen peroxide, while peroxidase catalyzed the oxidation of 4-aminoantipyrine by hydrogen peroxide and the reaction with coupled phenols to produce colored compounds with characteristic absorption peaks, and the glucose content was ultimately quantified by measuring the absorbance at 505 nm. Under acidic conditions, fructose reacts with resorcinol to produce a colored substance with a characteristic absorption peak, and its absorbance was measured at 480 nm to quantify the fructose content. Alkali was used to co-heat with the sample to destroy the reducing sugar in it. Sucrose was then hydrolyzed to glucose and fructose under acidic conditions, and then fructose reacted with resorcinol to produce a colored substance, which had a characteristic absorption peak at 480 nm, and the sucrose content was quantified by measuring the absorbance.

### 2.9. Statistical Analysis

All data were collected from three grape bunches, meaning that three separate bunches of grapes were measured for each set of samples. All data expressed as mean ± standard deviation. Experimental data were analyzed using IBM SPSS Statistics 27 software. One-way ANOVA and Duncan’s test were used to determine whether there was a statistical difference, and *p* < 0.05 was considered significant. Plotting was done using Origin 2024 software.

## 3. Results and Discussion

### 3.1. Analysis of Indicators Related to Postharvest Hardness of Grape Berries

**Figure 1:**
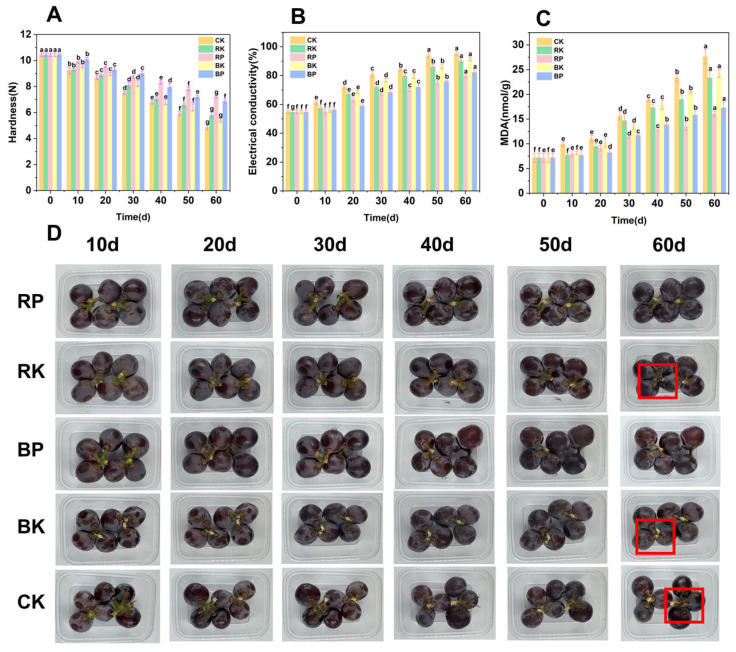
Changes in grape berry hardness (**A**), electrical conductivity (**B**), malondialdehyde (MDA) content (**C**), and representative image of grape samples (**D**) during storage under light treatment combined with modified atmosphere packaging. The CK group underwent sunlight-proofing; the RK group had continuous red LED illumination (660 nm); the RP group combined continuous red LED irradiation (660 nm) with modified atmosphere packaging; the BK group had continuous blue LED illumination (450 nm); and the BP group combined continuous blue LED irradiation (450 nm) with modified atmosphere packaging. The red areas indicated obvious browning of the fruit stalks. Different lowercase letters (a–g) indicated significant differences within groups (*p* < 0.05).

**Figure 2:**
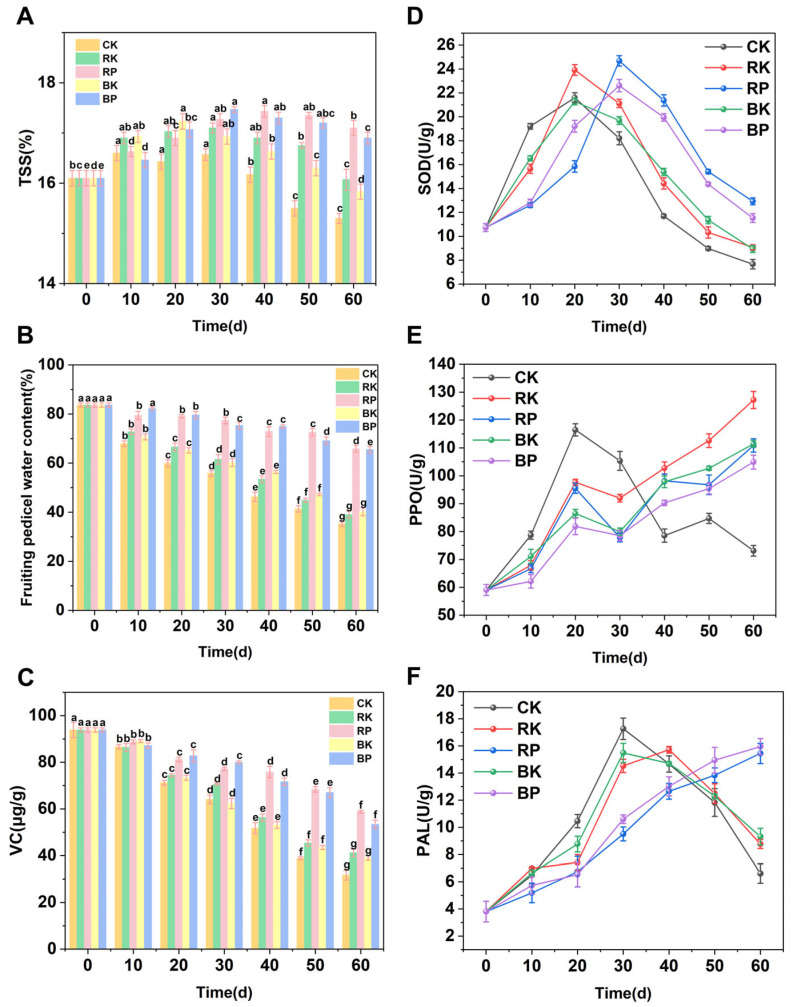
Changes in TSS (**A**), Fruiting pedicel water content (**B**), VC (**C**), SOD (**D**), PPO (**E**) and PAL (**F**) enzyme activity of grapes during storage under light treatment combined with modified atmosphere packaging. The CK group underwent sunlight-proofing; the RK group had continuous red LED illumination (660 nm); the RP group combined continuous red LED irradiation (660 nm) with modified atmosphere packaging; the BK group had continuous blue LED illumination (450 nm); and the BP group combined continuous blue LED irradiation (450 nm) with modified atmosphere packaging. Different lowercase letters (a–g) indicated significant differences within groups (*p* < 0.05).

Hardness is an important indicator of the postharvest quality and freshness of grapes, directly influencing their taste and storability. Hardness can objectively show the texture of postharvest grapes. Cell membrane permeability is also closely related to fruit firmness and carbohydrate metabolism. The cell membrane plays a vital role in carbohydrate metabolism in fruits, and improving its permeability can enhance postharvest carbohydrate metabolism [31,32,33]. A physical drawing of a grape is shown in Figure 1D. With the increase in storage time, the color of the fruit deepened slightly. This may be due to the continuous accumulation of anthocyanins under light conditions [34]. But this color change is not obvious. In Figure 1A, with extended storage time, the hardness of the grapes gradually decreased. The CK group showed the most significant decrease in hardness, with a decrease of 53.4%. To further investigate the causes of changes in grape hardness, the MDA content and cell membrane permeability of grape cells during storage were evaluated. In Figure 2B, the electrical conductivity of grapes in all groups increased over time, with the CK group exhibiting the largest increase in relative conductivity. Electrical conductivity is an indirect indicator of cell membrane integrity. The higher the electrical conductivity value, the more severe the cell membrane damage [35]. After 60 days of storage, the relative conductivity increased by 40.6% (*p* < 0.05), demonstrating significantly greater elevation compared to control and other treatment groups. This indicated that light treatment effectively inhibited the increase in relative conductivity in grapes. It was consistent with the results of Sanusi et al. [36]. After 60 days of storage, the relative conductivity of the RP and BP treatment groups was 79.7% and 82.2%, respectively. The relative conductivity of the CK, RK, and BK groups was 95.3%, 90.1%, and 91.5%, respectively. The relative conductivity of grapes in the RP and BP treatment groups increased by only 24.9% and 27.4%, respectively, which was significantly lower than that of the other treatment groups (*p* < 0.05). Furthermore, the relative conductivity in the RP group was slightly lower than that of the BP group after 30 days of storage (*p* > 0.05). Figure 2C showed the changes in MDA content of grapes during storage. MDA content is a quantitative marker of membrane lipid peroxidation. The higher the MDA content, the more serious the oxidative damage to the cell membrane [37]. It was observed that the MDA content of all grape groups increased over time. The MDA content was 7.18 nmol/g at day 0 and rose to 27.6 nmol/g after 60 days in the CK group, which was 15.6% and 12.3% higher than that in the RK and BK groups, respectively (*p* < 0.05). The MDA content in the RP and BP groups remained relatively low throughout the storage period, with levels of 11.3 nmol/g and 11.7 nmol/g, respectively. After 30 days, these values were consistently lower than those of the comparator groups (*p* < 0.05). This indicated that modified atmosphere packaging further reduced electrolyte leakage and alleviated cell membrane damage [38]. Additionally, after 50 days of storage, the MDA content of the RP group was significantly lower than that of the BP group (*p* < 0.05). The trends in conductivity, MDA content, and hardness of grapes during storage were negatively correlated (Figure 1B,C). The hardness values of 7.1 N and 6.7 N in the RP and BP groups, respectively, were still maintained after 60 days of storage, which were 48.4% and 40.7% higher than those of the CK group (*p* < 0.05). Furthermore, the hardness of grapes in the RP group was significantly higher than that in the BP group after 50 days of storage (*p* < 0.05). These results indicated that the combined treatment of light and modified atmosphere packaging significantly improved the quality of grapes during storage.

### 3.2. Impact of Light Treatment Combined with MAP on Postharvest Fruit Quality

TSS content and water content of the fruit pedicel are important physicochemical indicators of grape quality [39]. As shown in Figure 2A, the TSS content of grapes exhibited dynamic changes during storage. In the CK group, TSS levels fluctuated and then significantly decreased to 15.3% by day 60 (*p* < 0.05), which was markedly lower than in all other treatment groups. This indicated that red and blue light irradiation effectively reduced the degradation of TSS in stored grapes. These findings were consistent with previous research on postharvest grape preservation, where similar light treatments helped stabilize soluble solids content [40]. After a slight initial increase, the TSS content in both the RK and RP groups gradually declined during storage. Notably, the RK group maintained slightly higher TSS levels than the BK group after 30 days of storage. These observations suggested that red light treatment may be more effective than blue light in preserving grape TSS content during storage. This could be attributed to red light’s greater ability to elevate the gene expression levels of sucrose phosphorolytic synthase and invertase [26]. Furthermore, from day 40 onward, both RP and BP groups showed significantly higher TSS retention than the other groups (*p* < 0.05). While both treatments were effective in maintaining TSS content, the RP group consistently had slightly higher levels than the BP group throughout the storage period. These results clearly demonstrated that the combined light and modified atmosphere packaging treatments (RP and BP) were particularly effective in preserving grape TSS content during storage.

The fruiting pedicel is the physiologically active part of the grape berry and is also the primary site of water loss after harvest. By studying the water content of the pedicel, the freshness of the rachis could be indirectly reflected. Figure 2B illustrated the water loss in the fruit stalks across different treatment groups during the storage period [41]. Moisture loss occurred most rapidly in the CK group, with a 48.5% reduction in fruiting pedicel water content after 60 days of storage. This loss was significantly higher compared to the RK and BK groups (*p* < 0.05), highlighting the effectiveness of monochromatic light irradiation in reducing water loss from grape fruiting pedicels. Histological changes likely contributed to this phenomenon, including enhanced vascular differentiation and increased lignification of the xylem vessels within the fruiting pedicel’s vascular bundles [42]. These structural improvements would promote more efficient water transport and retention. The combined treatment groups (RP and BP) demonstrated particularly stable moisture retention, with final water loss rates of only 17.8% and 18.1%, respectively. Although no significant difference was found between these two groups (*p* > 0.05), both showed significantly better water retention compared to the CK group, with improvements of 30.7% and 30.3%, respectively (*p* < 0.05). These results clearly indicated that the combination of light irradiation and modified atmosphere packaging significantly reduced fruiting pedicel desiccation and effectively delayed browning during extended storage.

### 3.3. Impact of Light Treatment Combined with MAP on Postharvest Fruit Antioxidant Capacity

Figure 2C illustrates the changes in VC content of grapes, showing a decline in antioxidant capacity as storage time increased. The trend of VC content was inversely correlated with the increase in MDA content over time (Figure 1C), further confirming this relationship. The VC content in all groups decreased. The VC content was 31.7 μg/g after 60 days of storage in the CK group, a reduction of 66.2% compared to day 0, and 23.1% and 18.7% lower than the VC content in the RK and BK groups, respectively (*p* < 0.05). The VC content in the RP group decreased by only 37.3% after 60 days of storage, which was higher than in the other groups (*p* < 0.05). This indicated that the combined treatment of red light and MAP was more effective in preserving the VC content of grapes during storage, compared to red and blue light alone. This could be attributed to the fact that modified atmosphere packaging effectively maintained the VC content of grapes during storage [43]. As shown in Figure 2D, SOD activity in grapes under all treatments followed a characteristic pattern, initially increasing before gradually declining during storage. The RK group showed significantly higher SOD activity than the CK and BK groups after 20 days of storage (*p* < 0.05). From day 20 onward, both the RK and BK treatment groups maintained higher SOD activity compared to the CK group significantly (*p* < 0.05), indicating that monochromatic light treatments effectively enhanced the activity of this key antioxidant enzyme, improving oxidative stress resistance and delaying senescence [44]. Notably, the combined treatment groups (RP and BP) showed a delayed peak in SOD activity, reaching maximum values of 22.6 U/g and 24.7 U/g, respectively, at 30 days of storage—approximately 10 days later than the other treatment groups. After this peak, enzymatic activity gradually declined. Importantly, the RP-treated group maintained significantly higher SOD activity compared to all other treatments after 30 days of storage (*p* < 0.05). These results showed that the combination of red light and modified atmosphere packaging (RP treatment) most effectively boosted SOD activity, significantly enhancing the antioxidant capacity of stored grapes. This increased enzymatic protection helped reduce oxidative damage and better-preserved fruit quality [45]. In Figure 2E, the PPO activity of grapes in the CK group peaked at 116.5 U/g by day 20 of storage, representing a 2-fold increase from initial values, and remained higher than in other treatments until day 30 (*p* < 0.05). Both RK and BK treatments showed a steady increase in PPO activity throughout storage, reaching 2.2-fold and 1.9-fold of initial values, respectively. The RK group maintained significantly higher PPO activity than the BK group (*p* < 0.05). This result suggested that blue light was more effective than red light in suppressing PPO activity. The BP and RP treatments showed final PPO activities of 104.9 U/g and 110.9 U/g, respectively. BP treatment consistently maintained lower PPO levels throughout storage, significantly lower than the RK and RP groups at both 40 days and 60 days (*p* < 0.05). The inhibition of PPO activity by blue light was due to high-intensity visible light treatment causing irreversible structural changes in PPO [46,47]. The results indicated that combined light and modified atmosphere treatments suppressed the increase in PPO activity and delayed fruit browning during early storage (<30 days), with blue light showing slightly superior efficacy compared to red light. The high concentration of CO_2_ in the light combined with modified atmosphere packaging group may also be the reason for improving the antioxidant capacity of grapes [48]. PAL is an important element in the phenylpropanoid metabolic pathway, a mechanism that helps protect plants. Triggered by environmental factors, downstream compounds such as total phenols and flavonoids are synthesized, enhancing the plant’s resistance [14]. From Figure 2F, it can be observed that the PAL activity in the CK group gradually increased to its peak value before 30 days of storage and then slowly decreased. The lower PAL activity in the treatment groups during the pre-storage period may be due to slower senescence and reduced oxidative stress in the samples exposed to continuous light, resulting in lower PAL activity during this phase [49]. The PAL activity in the RK group peaked at 15.7 U/g by day 40 during the later stages of storage, showing a 10-day delay compared to the CK group, before gradually declining. RK maintained significantly higher PAL activity than the CK group after 60 days (*p* < 0.05), suggesting that red light treatment helped alleviate early oxidative stress, delay senescence, and sustain elevated PAL activity, thereby enhancing stress resistance during the later stages of storage [14]. Both RP and BP treatments showed a steady increase in PAL activity throughout storage, with values higher than the CK group after 50 days significantly (*p* < 0.05). These results suggested that combined light and MAP treatments delayed fruit aging effectively and helped prolong the quality of stored grapes.

### 3.4. Improvement of Carbohydrate Metabolism-Related Enzyme Activities by Light Treatment Combined with MAP

**Figure 3:**
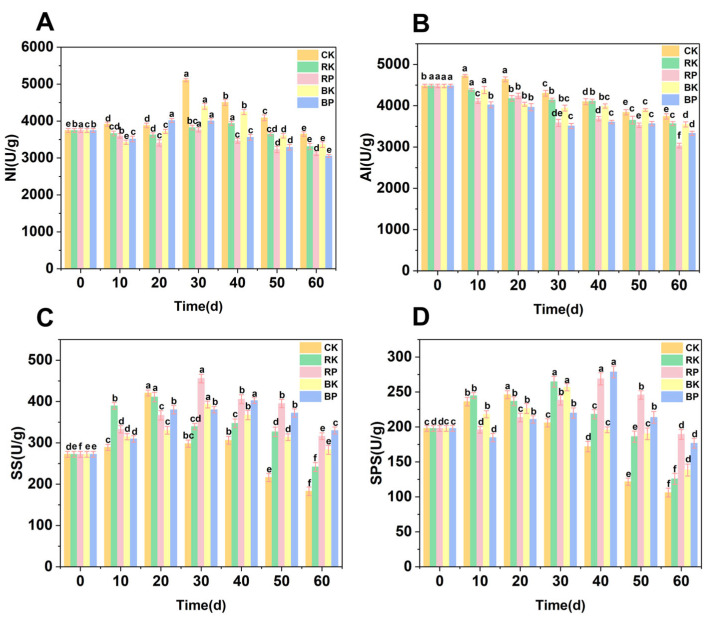
Changes in NI (**A**), AI (**B**), SS (**C**) and SPS (**D**) enzyme activity of grapes during storage under light treatment combined with modified atmosphere packaging. The CK group underwent sunlight-proofing; the RK group had continuous red LED illumination (660 nm); the RP group combined continuous red LED irradiation (660 nm) with modified atmosphere packaging; the BK group had continuous blue LED illumination (450 nm); and the BP group combined continuous blue LED irradiation (450 nm) with modified atmosphere packaging. Different lowercase letters (a–f) indicated significant differences within groups (*p* < 0.05).

**Figure 4:**
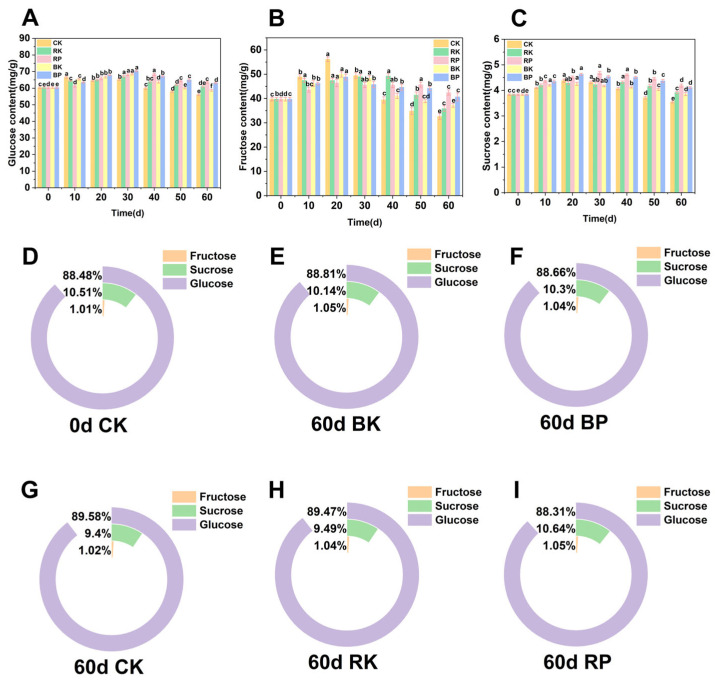
Changes in glucose content (**A**), fructose content (**B**), sucrose content (**C**) of grapes during storage under light treatment combined with modified atmosphere packaging. Percentage of sucrose content, fructose content, glucose content in the CK (**D**) group among the three sugar contents at 0 d of storage. Percentage of sucrose content, fructose content, glucose content in the BK (**E**), BP (**F**), CK (**G**), RK (**H**), RP (**I**) group among the three sugar contents at 60 d of storage. The CK group underwent sunlight-proofing; the RK group had continuous red LED illumination (660 nm); the RP group combined continuous red LED irradiation (660 nm) with modified atmosphere packaging; the BK group had continuous blue LED illumination (450 nm); and the BP group combined continuous blue LED irradiation (450 nm) with modified atmosphere packaging. Different lowercase letters (a–f) indicated significant differences within groups (*p* < 0.05).

As shown in Figure 3A, NI activity in CK grapes peaked at 30 days of storage before declining, maintaining significantly higher levels than in other treatments beyond this point. NI and AI, two key enzymes in plant carbohydrate metabolism, primarily catalyze the hydrolysis of sucrose into glucose and fructose. The RK and BK treatments significantly suppressed NI activity compared to the CK group during the 30–40 days. Notably, the RP treatment consistently showed lower NI activity than CK throughout storage (*p* < 0.05). The NI enzyme activity in the RP treatment group was 3406.5 U/g on day 20 and 3761.5 U/g on day 30. In the BP treatment group, the NI enzyme activity was 4016.7 U/g on day 20 and 4004.0 U/g on day 30. RP exhibited significantly reduced NI activity compared to BP between days 20 and 30 (*p* < 0.05) and outperformed both RK and BK after day 40 (*p* < 0.05). These findings clearly demonstrate that the combined red light and MAP treatment most effectively controlled NI activity, thereby better preserving sucrose content in stored grapes compared to other treatments [50]. Figure 3B illustrates the changes in AI enzyme activity of grapes across different treatment groups during storage. Prior to 40 days of storage, the AI activity in the RK and BK groups was reduced compared to the CK group (*p* < 0.05). The combined red and blue light modified atmosphere packaging (RP and BP) treatments demonstrated significantly lower AI enzyme activity compared to RK, BK, and CK groups at 10, 30, 40, and 60 days (*p* < 0.05). Sucrose synthase is a common enzyme in plants, primarily involved in the synthesis and metabolism of sucrose, but also plays a role in breaking down sucrose into glucose and fructose through a reverse catalytic process [51]. Sucrose synthase activity increased rapidly in the CK group after 10 days, which was due to the need for rapid energy supply due to environmental stress and other factors (Figure 3C). SS enzyme provides energy for grapes to support their normal metabolic activities by participating in the catabolic reaction of sucrose and using stored sucrose. After 30 days, the SS enzyme activity in the RK and BK groups was significantly higher than that of the CK group (*p* < 0.05), suggesting that the red and blue light treatments could effectively increase the SS enzyme activity and promote sucrose synthesis [52]. RP and BP groups were significantly higher than the remaining three groups after 40 days of storage (*p* < 0.05), indicating that light-MAP combination treatments were more effective in slowing down the degradation of sucrose. SPS is a key enzyme in plants that plays a crucial role in the biosynthesis of sucrose [53]. Figure 3D illustrates the temporal changes in SPS activity across treatment groups during storage. Both RK and BK groups exhibited significantly higher SPS activity than CK after 20 d (*p* < 0.05), demonstrating that monochromatic light treatments enhanced sucrose synthesis and maintained fruit energy supply through SPS activation in later storage stages. This effect may be attributed to light-induced upregulation of SPS-related genes (MrSPS1, MrSPS2, and MrSPS3) [54]. The RP and BP groups were significantly higher (*p* < 0.05) than the CK, RK and BK groups after 40 days, where the RP group was significantly higher than the BP group at 30 and 50 days. Suggesting that the red light modified atmosphere packaging treatment was more effective in increasing the SPS enzyme activity and thus maintaining the sucrose content during the storage period as compared to the blue light combined modified atmosphere packaging treatment (Figure 4B).

### 3.5. Relationship Between Changes in Sugar Content and Activities of Carbohydrate Metabolism-Related Enzymes in Postharvest Grape Berries

Changes in glucose content of grapes during storage are shown in Figure 4A. The CK group showed a rapid increase within 10 days of storage, which may be related to the higher AI enzyme activity in the pre-storage period that promoted the decomposition of sucrose into glucose (Figure 3B). 60 days after storage, the glucose content was 31.08 mg/g, which was significantly lower than that of the RK and BK groups (*p* < 0.05). The glucose content of the RK group was slightly lower than that of the BK group before 30 days. But the glucose content of the RK group was 34.34 mg/g, which was significantly higher than that of the BK group after 50 days of storage (*p* < 0.05). The glucose content of RP and BP treatments was significantly greater than that of other treatment groups after 40 days (*p* < 0.05). Figure 4B showed the changes in fructose content in each group. It can be observed that the fructose content of the CK group increased to 56.15 mg/g at 20 days of storage, which may be due to the increase in the activities of the AI and NI enzymes that accelerated the conversion of sucrose to glucose and fructose (Figure 3A,B) [55]. The fructose content of RK treatment group remained 49.23 mg/g at 40 days of storage, which was significantly higher than that of BK and CK groups (*p* < 0.05). The BK group was significantly higher than that of CK group after 40 days (*p* < 0.05). During the storage period, the fructose content of the RP and BP groups changed more gently. At the end of storage, the fructose contents of 42.48 mg/g and 40.66 mg/g were still maintained, which were significantly higher than those of CK, RK and BK groups. In addition, the fructose content of RP group was slightly higher than that of blue light after 30 days of storage. Sucrose is one of the major sugars in grapes, which serves as a form of energy storage in the fruit and maintains the energy required during post-ripening [56]. Figure 4C showed the variation in sucrose content during storage in grapes. It can be found that the sucrose content of each group firstly increased and then decreased during the storage period. The sucrose content of the CK group increased to 4.38 mg/g at 20 days of storage and then began to gradually decline, and was 3.54 mg/g at 60 days of storage, which was significantly lower than the other treatment groups (*p* < 0.05). This indicated that red light and blue light treatments can effectively slow down the decline of sucrose content in grapes. This can be attributed to the effect of light on the enzyme activity of AI and NI [57,58]. The sucrose content of grapes in the RP group was significantly higher than that of the CK, RK and BK groups during the storage period (*p* < 0.05). Suggesting that red light combined with MAP treatment maintained grape sucrose content significantly, which may be due to the increase in SS and SPS enzyme activities (Figure 3C,D). In addition, red light treatment was found to increase sucrose content and SPS enzyme activity in other similar studies [59]. The sucrose content in the CK group decreased by day 60 of storage compared to day 0 (Figure 4D,G). After 60 days of storage, the sucrose content in the RP and BP treatment groups was 10.64% and 10.3%, respectively (Figure 4F,I), which was higher than that in the other treatment groups (Figure 4E,G,H). This demonstrates that sucrose decomposition can be effectively alleviated by the combination of red light and modified atmosphere packaging treatment.

## 4. Conclusions

This research demonstrated that red and blue light combined with modified atmosphere packaging treatment significantly improved the quality of Kyoho grapes during storage. This research primarily investigated the physicochemical properties and carbohydrate metabolism of Kyoho grapes. The grapes of all groups maintained a consistently good appearance and color during the study period. The combination of MAP and LED is primarily an additive effect. The photos of grape sample showed that there was no visible mold on the surface of the grapes stored for 60 days, indicating that the grapes were not contaminated by microorganisms during this period. The hardness, VC content and TSS content of postharvest grapes can be used as quantitative indicators to objectively evaluate their texture and taste. It showed that the taste and texture of grapes were well maintained within 60 days under MAP combined with LED packaging. Light combined with MAP significantly improved the antioxidant capacity of fruits by increasing the activity of SOD and PAL enzymes. Light combined with MAP effectively regulated the carbohydrate metabolism of grape berries and significantly slowed down the loss of sugar content. Light combined with MAP effectively maintained the activity of SS and SPS in the late stage of grape storage, thus slowing down the loss of sucrose content. After 60 days of storage, the glucose, fructose, and sucrose contents of the RP group increased by 13.4%, 30.2%, and 18.1%, respectively, compared to the CK group (*p* < 0.05). As a result, red light combined with MAP extended the shelf life of grapes up to 60 days. In addition, future research would give priority to odor evaluation, so as to build a more perfect comprehensive evaluation system of grape postharvest quality.

## Figures and Tables

**Table 1:** Overview of the experimental design.

Samples	Treatment
CK	sunlight-proofing
RK	Continuous red LED illumination (660 nm)
RP	Continuous red LED irradiation (660 nm) and Modified atmosphere packaging
BK	Continuous blue LED illumination (450 nm)
BP	Continuous blue LED irradiation (450 nm) and Modified atmosphere packaging

## Data Availability

The original contributions presented in the study are included in the article. Further inquiries can be directed to the corresponding authors.
